# Gene Expression of Human Lung Cancer Cell Line CL1–5 in Response to a Direct Current Electric Field

**DOI:** 10.1371/journal.pone.0025928

**Published:** 2011-10-05

**Authors:** Ching-Wen Huang, Huai-Yi Chen, Meng-Hua Yen, Jeremy J. W. Chen, Tai-Horng Young, Ji-Yen Cheng

**Affiliations:** 1 Institute of Biomedical Engineering, National Taiwan University, Taipei, Taiwan; 2 Research Center for Applied Sciences, Academia Sinica, Taipei, Taiwan; 3 Department of Engineering and System Science, National Tsing-Hua University, Hsinchu, Taiwan; 4 Nano Science and Technology Program, Taiwan International Graduate Program (TIGP), Academia Sinica, Taipei, Taiwan; 5 Institutes of Biomedical Sciences and Molecular Biology, National Chung-Hsing University, Taichung, Taiwan; 6 Department of Mechanical and Mechantronic Engineering, National Taiwan Ocean University, Keelung, Taiwan; 7 Institute of Biophotonics, National Yang-Ming University, Taipei, Taiwan; Tel Aviv University, Israel

## Abstract

**Background:**

Electrotaxis is the movement of adherent living cells in response to a direct current (dc) electric field (EF) of physiological strength. Highly metastatic human lung cancer cells, CL1–5, exhibit directional migration and orientation under dcEFs. To understand the transcriptional response of CL1–5 cells to a dcEF, microarray analysis was performed in this study.

**Methodology/Principal Findings:**

A large electric-field chip (LEFC) was designed, fabricated, and used in this study. CL1–5 cells were treated with the EF strength of 0mV/mm (the control group) and 300mV/mm (the EF-treated group) for two hours. Signaling pathways involving the genes that expressed differently between the two groups were revealed. It was shown that the EF-regulated genes highly correlated to adherens junction, telomerase RNA component gene regulation, and tight junction. Some up-regulated genes such as *ACVR1B* and *CTTN*, and some down-regulated genes such as *PTEN*, are known to be positively and negatively correlated to cell migration, respectively. The protein-protein interactions of adherens junction-associated EF-regulated genes suggested that platelet-derived growth factor (PDGF) receptors and ephrin receptors may participate in sensing extracellular electrical stimuli. We further observed a high percentage of significantly regulated genes which encode cell membrane proteins, suggesting that dcEF may directly influence the activity of cell membrane proteins in signal transduction.

**Conclusions/Significance:**

In this study, some of the EF-regulated genes have been reported to be essential whereas others are novel for electrotaxis. Our result confirms that the regulation of gene expression is involved in the mechanism of electrotactic response.

## Introduction

Electrotaxis, also known as galvanotaxis, is the movement of organisms or cells in response to an electric field (EF). Physiological EF exists extracellularly, such as in wound, embryo's skin, and ducts [1], [2]. It may also exist in the interface between tumors and normal tissues [3], [4]. Several types of cancer cells such as prostate cancer cells, breast cancer cells, and lung cancer cells, are known to migrate directionally under dcEF, and the degree of electrotaxis of these cancer cells has been shown to correlate to their metastatic abilities [5]–[8]. Living cell electrotaxis is different from cell electrophoresis since the latter requires the detachment of cultured cells from the substrate in advance [9]. In addition, the EF strength for cell electrophoresis is about 100-fold higher than that for electrotaxis [9]. Also, it has been shown that the directional migration of the cells was caused by EFs but not EF-induced events such as electro-osmotic flows [10].

The mechanism of electrotaxis has been explored for more than 10 years. Several important proteins and genes are reported to be involved. It is known that physiological EF redistributes epidermal growth factor receptors (EGFRs), leading to the cathodal polarization and the activation of EGFR-mitogen-activated protein kinase (MAPK) signaling pathway [11], [12]. EGFR signaling is essential for EF-directed migration of breast cancer cells [6]. Besides, cyclic AMP (cAMP) and cAMP-dependent protein kinase A mediate the directional migration of human keratinocytes in a dcEF [13], [14]. Beta-4 integrin together with epidermal growth factor (EGF) also mediate the electrotaxis of human keratinocytes through a Rac-dependent signaling pathway [15]. Electrical signals control wound healing through phosphatidylinositol-3-OH kinase (PI3K)-γ and phosphatase and tensin homolog (PTEN) [16]. The EF stimulation triggers the activation of Src and inositol–phospholipid signaling, which polarizes in the direction of the epithelial cell migration [16]. In *Xenopus* embryonic spinal neurons, GTPases Cdc42, Rac and Rho mediate growth cone steering in a physiological EF through the spatiotemporal regulation of GTPase activity and their effectors [17], [18]. The EF-guided migration of rat hippocampal neurons is mediated by the activation of Rho-associated protein kinase (ROCK) and PI3K [19]. In addition, the direction of the EF-induced migration of *Dictyostelium* cells is switched by cGMP and phosphatidylinositol signaling [20]. It is also reported that the calcium ions play important roles in the directed migration of *Dictyostelium* cells and the osteoblast-like cells [21], [22], suggesting that calcium signaling pathway may participate in the electrotaxis.

Most studies exploring the mechanism of electrotaxis are focused on specific genes, proteins, and signaling pathways. The global gene expression profiling could be an approach to reveal the whole view of the mechanism. DNA microarray is a well-known technology for genome-wide gene expression profiling. Recently, the microarray analysis for electrotaxis study has been performed in human dermal fibroblasts and epidermal keratinocytes [23], [24]. In human dermal fibroblasts, the EF-regulated genes are associated with cellular signaling pathways including TGF-β, G-proteins, and inhibition of apoptosis [23]. In human epidermal keratinocytes, the EF-regulated genes are shown to correlate with chemokine, apoptosis, JAK-STAT, Wnt, and G-protein MAPK activation signaling pathways [24]. So far, there is no research work discussing the global effect of EF on the gene expression of cancer cells.

Tumor cell invasion and metastasis are the major causes resulting in the high mortality of lung cancer patients in five years. Invasion is the most critical step in the metastatic process and it occurs through the interaction between the tumor cells and the surrounding environment. Human lung adenocarcinoma cells, CL1-5, which is a sub-line derived from CL1-0, has higher invasiveness than CL1-0 [25]. In our previous study, we have shown that CL1-5 cells migrate toward the anode and orient perpendicularly to the direction of the dcEF. In contrast, CL1-0 did not show obvious electrotactic response [8]. Since the positive correlation between the metastatic ability and the electrotactic response has been observed in the level of cell motion, it is important to further investigate the influence of physiological EF on the gene expression. In this work, the highly metastatic CL1–5 cells were examined by using DNA microarray. Through the analysis of the EF-regulated genes and their corresponding signaling pathways, we may understand more about the role of physiological EF in tumor metastasis.

**Figure 1:**
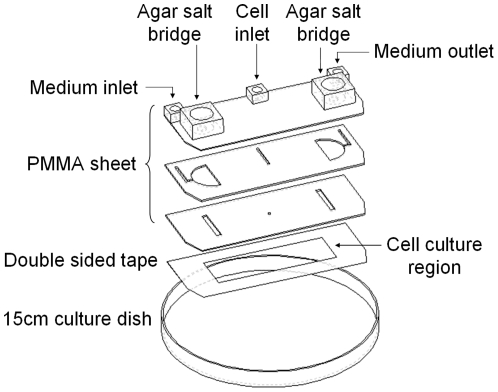
Assembly drawing of the large electric-field chip (LEFC). The LEFC had connecting holes for the medium inlet/outlet and the agar salt bridges. Cells were cultured in the micro-chamber (the cell culture region). The width, length, and thickness of the micro-chamber were 24mm, 75mm, and 70 µm, respectively.

For the electrotaxis study, we have designed and fabricated a microfluidic electric-field chip (EFC) which provides uniform dcEF in the cell culture micro-chamber [8]. The thickness of the micro-chamber is only 70 µm and thus the joule heating can be omitted [8]. The limitation of the EFC is that the cell culture region is too small to provide enough cells for microarray analysis in one-time experiment. Therefore, a large electric-field chip (LEFC) providing uniform dcEF was designed and fabricated in this work for sample collection (Figure 1).

## Results

### LEFC and EF stimulation

**Figure 2:**
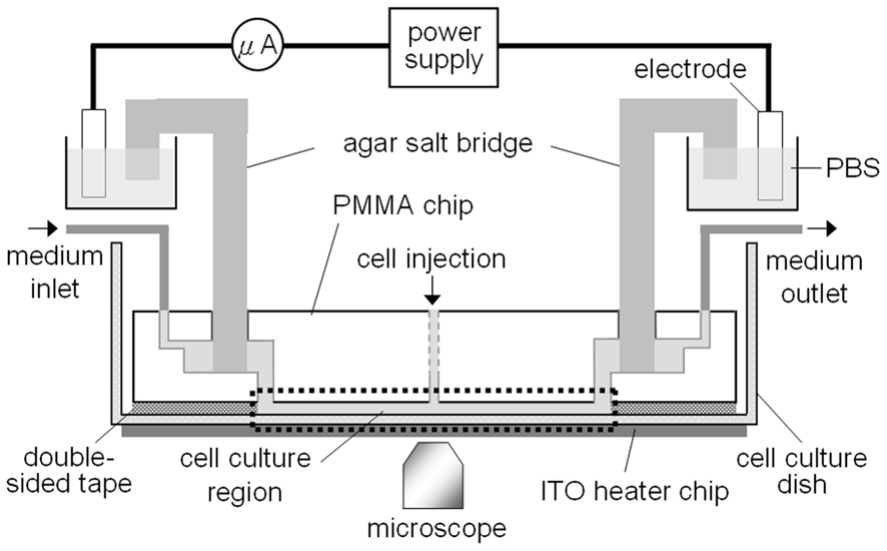
Lateral view of the electrotaxis system. A LEFC was integrated with a transparent ITO heater chip, two Ag/AgCl electrodes with phosphate-buffered saline (PBS) as electrolyte, two agar salt bridges (1.5% agar in PBS), a syringe pump, a DC power supply, an ampere-meter, and an inverted microscope.

**Figure 3:**
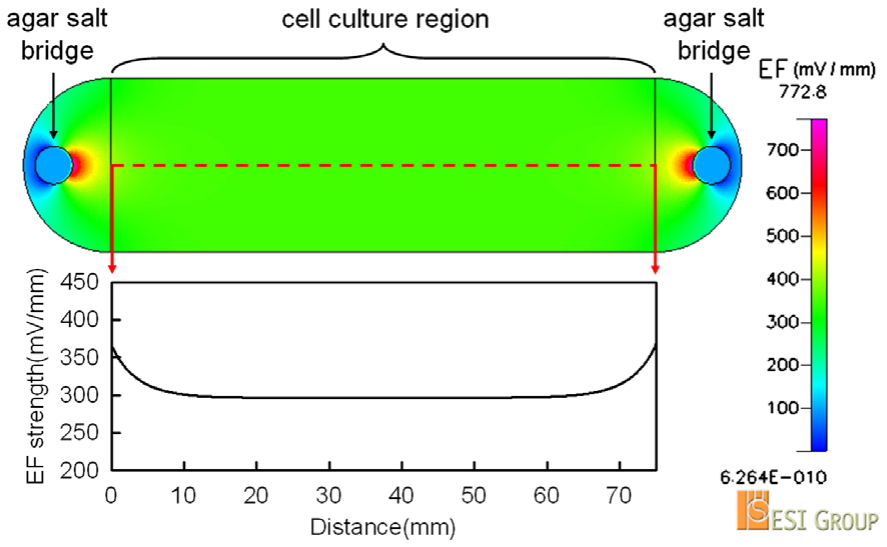
Simulated EF in the cell culture region of the LEFC. The EF strength along the dotted line (between the two arrows) was shown. More than 85% culture area was exposed to the EF strength of 300+/−15mV/mm.

To build up an EF with the strength of 300mV/mm in the cell culture region, the current flow of 696 µA was introduced into the LEFC by applying the voltage of about 21V on the electrodes (Figure 2). The electrical power consumed in the cell culture region was estimated to be P =  IV  = 15.7mW (696 µA×75mm×300mV/mm). It was expected that joule-heating could be omitted with such low electrical power. The numerical simulation of the dcEF showed a uniform distribution in the cell culture region (Figure 3). More than 85% culture region was exposed in the EF strength of 300+/−15mV/mm.

In microarray analysis, 20–30 µg total RNA is required for one GeneChip, which means that about 10^6^ CL1–5 cells are needed for each replicate. The cell culture region of a LEFC is 75×24mm^2^, about 40-fold larger than that of an EFC (3×15mm^2^). It was tested that CL1–5 cells harvested from a LEFC could reach to 10^6^ cells per chip. In other words, a LEFC could provide enough amount of total RNA for one microarray experiment.

### Signaling pathway analysis

Through the ANOVA test, 1631 probe sets of Affymetrix HG-U133 plus2.0 Array were identified with statistically differential expression between the control group and the EF-treated group (p<0.05). Among them, 431 probe sets had all signal intensities of the two groups larger than the global median intensity of the six arrays ( = 66.5, from the signal intensities of 54675 probe sets×3 replicates×2 groups). The 431 probe sets were considered in the signaling pathway analysis. The accession numbers of these probe sets were submitted to CRSD website, where the genome-wide iterative enrichment analysis was performed. The top three signaling pathways showing significant correlation to the EF-regulated genes were adherens junction, telomerase RNA component gene *hTerc* transcription regulation, and tight junction (Table 1).

**Table 1:** Signaling pathways which were significantly correlated to the EF-regulated genes.

Signaling Pathway	Accession #	Gene Symbol	Gene Name	Fold Change
Adherens junction (KEGG)	NM_020328	*ACVR1B*	Activin A receptor, type IB	1.51
	M14333	*FYN*	FYN oncogene related to SRC, FGR, YES	1.21
	AB020707	*WASF3*	WAS protein family, member 3	1.2
	BG035989	*ACP1*	Acid phosphatase 1, soluble	1.18
	AW139723	*PVRL1*	Poliovirus receptor-related 1 (herpesvirus entry mediator C; nectin)	1/1.97
	NM_001614	*ACTG1*	Actin, gamma 1	1/1.05
Telomerase RNA component gene hTerc Transcriptional Regulation (BioCarta)	BF445142	*NFYA*	Nuclear transcription factor Y, alpha	1/1.16
	NM_014223	*NFYC*	Nuclear transcription factor Y, gamma	1/1.27
Tight junction (KEGG)	AF191495	*F11R*	F11 receptor	1.52
	BG475299	*CTTN*	Cortactin	1.51
	NM_002870	*RAB13*	RAB13, member RAS oncogene family	1.25
	N63821	*EPB41*	Erythrocyte membrane protein band 4.1 (elliptocytosis 1, RH-linked)	1.23
	AK024986	*PTEN*	Phosphatase and tensin homolog (mutated in multiple advanced cancers 1)	1/1.19
	NM_001614	*ACTG1*	Actin, gamma 1	1/1.05

### Subcellular localization and biological function analysis

The EF-regulated genes were categorized based on the subcellular location of their protein products. The protein database Swiss-Prot and Ensembl were referred to. If a protein was located in “cell membrane” shown in Swiss-Prot, or it contained “signal peptide and transmembrane domain” shown in Ensembl, it was categorized as a cell membrane protein in this study. Based on the definition, 6545 probe sets of HG-U133 Plus2.0 array were considered to represent the genes encoding cell membrane proteins. Here we examined the significantly regulated genes with the fold change larger than 1.5 or smaller than 1/1.5 (the EF-treated group/the control group). Among 88 genes which met the criteria, 18 genes were recognized to encode cell membrane protein. The percentage is 18.2% ( = 16/88). For comparison, we randomly selected 88 probe sets from the probe sets of HG-U133 plus2.0 array (excluding the probe sets of control sequences) and examined the percentage of genes encoding cell membrane protein. The average percentage from 10000-time operation is 12%.

**Figure 4:**
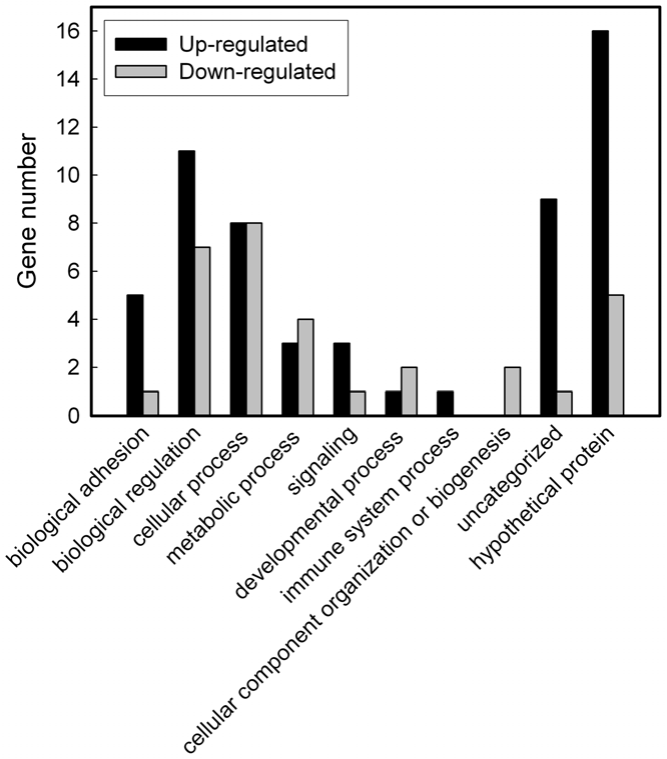
Biological processes correlated with the EF-regulated genes. The significantly regulated genes listed in Table 2 (up-regulated) and Table 3 (down-regulated) were categorized with their biological function according to the Gene Ontology annotation.

The categorization of the 88 significantly regulated genes was shown in Table 2 (up-regulated) and Table 3 (down-regulated). Apart from the genes encoding cell membrane proteins, others were categorized based on the “cellular component” in Swiss-Prot. In addition, the genes listed in Table 2 and Table 3 were analyzed with their biological function according to the Gene Ontology annotation (Figure 4). The microarray analysis result was partially validated by real-time RT-PCR. The 20 selected genes showed positive correlation between the real-time RT-PCR and the microarray analysis results in their expression change (Table 2 and 3).

**Table 2:** Significantly up-regulated (>1.5-fold) genes by dcEF.

Gene Name	Gene Symbol	Accession #	Fold change	
			Microarray	Real-time PCR
**Cell Membrane**				
Transmembrane 6 superfamily member 1	*TM6SF1*	NM_023003	1.83	1.22
Major histocompatibility complex class I-related gene protein	*MR1*	AF031469	1.81	1.59
Neurotrimin	*NTM*	AW085558	1.8	1.28
5′-3′ exoribonuclease 1	*XRN1*	BC039314	1.72	
Integrin beta-5	*ITGB5*	NM_002213	1.55	
Coagulation factor II (thrombin) receptor	*F2R*	BG026194	1.53	
Junctional adhesion molecule A, F11 receptor	*F11R*	AF191495	1.52	
Activin receptor type-1B	*ACVR1B*	NM_020328	1.51	
**Secreted**				
Stanniocalcin-1	*STC1*	U46768	1.78	1.49
Fibronectin type-III domain-containing protein C4orf31	*C4orf31*	NM_024574	1.72	1.49
Serglycin	*SRGN*	J03223	1.69	
Aspartylglucosaminidase	*AGA*	M64073	1.56	
Wingless-type MMTV integration site family, member 5A	*WNT5A*	NM_003392	1.54	
**Cytoplasm**				
Myristoylated alanine-rich C-kinase substrate	*MARCKS*	AA770596	1.77	
Aryl hydrocarbon receptor repressor	*AHRR*	AB033060	1.67	1.53
Notch homolog 2 N-terminal-like protein	*NOTCH2NL*	AW024960	1.61	
Enolase 3 beta	*ENO3*	NM_001976	1.6	1.22
E3 ubiquitin-protein ligase MIB2	*MIB2*	BE222279	1.6	
SEC14-like protein 2	*SEC14L2*	NM_012429	1.56	1.26
Desmoplakin	*DSP*	AW444944	1.52	
Src substrate cortactin	*CTTN*	BG475299	1.51	
**Nucleus**				
Neuroblast differentiation-associated protein AHNAK	*AHNAK*	NM_024060	2.05	1.76
Tumor protein p53-inducible nuclear protein 1	*TP53INP1*	AW341649	1.95	1.74
E3 SUMO-protein ligase PIAS2	*PIAS2*	AF077953	1.7	
Chromobox protein homolog 5	*CBX5*	BE568225	1.63	
Zinc finger protein 232	*ZNF232*	AW173312	1.6	1.27
Splicing factor, arginine/serine-rich 11	*SFRS11*	AW241752	1.53	
Transducin-like enhancer protein 1	*TLE1*	BE302305	1.51	1.22
**Mitochondrion**				
Putative transferase C1orf69, mitochondrial	*C1orf69*	AW243177	1.53	
**Lysosome**				
Solute carrier family 29 (nucleoside transporters), member 3	*SLC29A3*	NM_018344	1.79	1.23
**Uncategorized**				
Polycomb group RING finger protein 3	*PCGF3*	BG231712	2.19	1.5
Next to BRCA1 gene 2 protein	*NBR2*	BC034248	2.05	1.85
Dedicator of cytokinesis protein 9	*DOCK9*	AW450751	1.94	
Probable protein phosphatase 1N	*PPM1N*	BE732320	1.88	
1-aminocyclopropane-1-carboxylate synthase-like protein 1	*ACCS*	AI676022	1.85	
Glutamate decarboxylase 1	*GAD1*	NM_013445	1.84	1.39
Solute carrier family 6 (neurotransmitter transporter, taurine), member 6	*SLC6A6*	BC006252	1.7	
Chromosome 11 open reading frame 71	*C11orf71*	AV721563	1.67	
Family with sequence similarity 167, member A	*FAM167A*	BE856336	1.6	
Kelch repeat and BTB domain-containing protein 11	*KBTBD11*	NM_014867	1.6	
Fibrinogen C domain-containing protein 1	*FIBCD1*	BF439289	1.56	1.2
Uncharacterized protein C17orf90	*C17orf90*	AY007126	1.56	
**Unknown (Hypothetical protein)**				
LOC100289219	*---*	AI188104	2.23	
cDNA clone IMAGE:3879939	*---*	BE789947	2.14	
cDNA clone IMAGE:2526201	*---*	AW024656	2.08	
cDNA clone IMAGE:1127501	*---*	AA653638	2.07	
LOC100132999	*---*	AA532655	1.73	
cDNA clone IMAGE:1336731	*---*	AA809353	1.71	
cDNA clone IMAGE:2976454	*---*	AW629461	1.71	
cDNA clone IMAGE:2062250	*---*	AI343467	1.69	
LOC84856 (hypothetical non-coding RNA)	*---*	AK024638	1.59	
cDNA clone IMAGE:2055268	*---*	AI307251	1.57	
cDNA clone cdAAME10	*---*	AV734194	1.56	
LOC100134259	*---*	W72564	1.53	
cDNA clone IMAGE:3233624	*---*	BE672607	1.53	
cDNA clone IMAGE:3285426	*---*	BE671045	1.52	
cDNA clone IMAGE:1946650	*---*	AI341823	1.51	

**Table 3:** Significantly down-regulated (< 1/1.5 fold) genes by dcEF.

Gene Name	Gene Symbol	Accession #	fold change	
			Microarray	Real-time PCR
**Plasma Membrane**				
Poliovirus receptor-related protein 1	*PVRL1*	AW139723	1/1.97	1/1.29
Heparan-sulfate 6-O-sulfotransferase 1	*HS6ST1*	BC001196	1/1.79	1/1.21
Armadillo repeat-containing protein 10	*ARMC10*	AY150851	1/1.78	
Beta-1,4-galactosyltransferase 6	*B4GALT6*	BG503479	1/1.69	
ATP-binding cassette sub-family A member 7	*ABCA7*	NM_019112	1/1.68	
PDZ and LIM domain 5	*PDLIM5*	AF116705	1/1.58	
G-protein coupled receptor 156	*GPR156*	AW451851	1/1.53	
Pro-neuregulin-2, membrane-bound isoform	*NRG2*	AI271427	1/1.52	
**Secreted**				
Latent-transforming growth factor beta-binding protein 3	*LTBP3*	AW515704	1/1.60	
**Cytoplasm**				
Methylosome protein 50 (WD repeat domain 77)	*WDR77*	NM_024102	1/1.81	
PAS domain-containing serine/threonine-protein kinase	*PASK*	U79240	1/1.81	
TNF receptor-associated factor 2	*TRAF2*	NM_021138	1/1.79	
SH3 and PX domain-containing protein 2A	*SH3PXD2A*	NM_014631	1/1.55	
Baculoviral IAP repeat-containing protein 7	*BIRC7*	NM_022161	1/1.51	
**Nucleus**				
REST corepressor 2	*RCOR2*	BF528119	1/1.76	
Kinetochore-associated protein NSL1 homolog	*NSL1*	AW168886	1/1.60	
**Mitochondrion**				
Mitochondrial ribosomal protein L38	*MRPL38*	AU143610	1/1.64	
Cardiolipin synthase 1	*CRLS1*	AI339837	1/1.58	
Long-chain-fatty-acid--CoA ligase 3	*ACSL3*	NM_004457	1/1.52	
**Uncategorized**				
Psoriasis associated RNA induced by stress (non-protein coding), non-coding RNA	*PRINS*	AK022045	1/1.94	
Phosphate cytidylyltransferase 2, ethanolamine	*PCYT2*	BC000351	1/1.89	1/1.37
Chromosome 11 open reading frame 31	*C11orf31*	AF085883	1/1.81	
Glutamate--cysteine ligase regulatory subunit	*GCLM*	NM_002061	1/1.63	
Rap guanine nucleotide exchange factor 1	*RAPGEF1*	AU158380	1/1.54	
Guanine nucleotide binding protein (G protein), alpha 11 (Gq class)	*GNA11*	AL110227	1/1.51	
Unc-5 homolog B (C. elegans)	*UNC5B*	AL049370	1/1.51	
**Unknown (Hypothetical protein)**				
cDNA clone IMAGE:2157199	*---*	AI476341	1/1.87	
cDNA clone IMAGE:1560745	*---*	AA969238	1/1.67	
cDNA clone GKCBWAO9	*---*	AV699953	1/1.65	
LOC401312	*---*	BC042871	1/1.63	
cDNA clone UI-CF-DU1-ado-n-20-0-UI 3-	*---*	BU687162	1/1.58	

### Gene expression of reported electrotaxis-related genes/proteins

We also examined the microarray analysis results of the genes that have been reported to be correlated to electrotaxis. Among the 1631 probe sets identified by ANOVA, those that have all intensities above the background level (i.e. larger than the global median) in at least one of the two groups were considered. The expression of electrotaxis-related genes/proteins was discussed below.

## Discussion

### EF stimulation

Under the EF strength of 300mV/mm, our previous work has shown that CL1–5 cells have a significant tendency of directional migration and orientation while CL1-0 cells move randomly [8]. CL1–5 cells present a nearly constant movement towards the anode immediately after the dcEF is turned on. However, the orientation of CL1–5 cells is not evident until 2-hour exposure in the dcEF. The different response time of the EF-induced directional migration and the cell body orientation of CL1–5 cells suggest the involvement of different signal pathways in electrotaxis. This point of view is also suggested in a recent work by Hammerick et al [26].

We presumed that the gene expression change associating with the two electrotactic responses, the directional migration and the orientation, could all be detected after 2-hour treatment of the dcEF. Thus, in this study the microarray analysis was performed for CL1–5 cells stimulated by 300mV/mm for 2 hours. The results showed that several genes were significantly regulated. Considering the high cost of DNA microarray analysis (about US$1,000×3 replicates×2 groups for each time point), we started by choosing the time period of 2 hours in this initial study. To further distinguish if the regulated gene was correlated to the directional migration or the orientation, microarray analysis of short term EF stimulation (perhaps 10mins) will be pursued in our future work.

In this study, the cell culture medium was replaced with serum-free DMEM medium before the dcEF was applied. Serum is a complex compound which contains different proteins like growth factors, cytokines, and attachment factors. Under an applied dcEF in the cell culture chamber, chemical gradient of these proteins may be generated by electrophoresis. Therefore, the response of cells under the dcEF might be affected by chemotaxis. To minimize the possible effects of the chemical gradient when investigating the influence of dcEF on the cells, the serum was removed in advance. Our previous work has shown that the dcEF induces the directional migration and the orientation of CL1–5 cells in serum-free medium [8]. The results suggest that the dcEF could induce the electrotactic response of living cells without chemical stimulation. Some different types of cells also show electrotactic response in serum-free medium, such as neural crest cells, hippocampal neurons, and CHO cells [27]–[29].

Nevertheless, cells are surrounded by a variety of growth factors and cytokines *in vivo* which may also play roles in the electrotaxis of cells. In our previous study, CL1–5 cells have been observed to migrate toward the anode both in serum-free [8] and in serum-containing (Movie S1) medium under an applied dcEF. It is an important future work to compare the EF-regulated gene expression in serum-containing medium and that in serum-free medium. The concurrent effect of the EF and the chemical gradient could thus be investigated. Such work may help us to find out the candidate growth factors and cytokines that participate in the mechanism of electrotaxis.

### Signaling pathway analysis

Under the treatment of the dcEF, we observed the differential regulation of six genes which were known to be involved in the adherens junction pathway (hsa04520, KEGG) (Table 1). Four genes *ACVR1B* (1.51-fold), *FYN* (1.21-fold), *WASF3* (1.2-fold), and *ACP1* (1.18-fold) were shown to be up-regulated. *ACVR1B* (activin A receptor, type IB) encodes a type I activin receptor, which is a transmembrane serine/threonine kinase receptor and is essential for signaling. Activin receptor signaling regulates prostatic epithelial cell adhesion and is associated with the prostate cancer metastasis [30]. The protein tyrosine kinase Fyn (encoded by *FYN, FYN* oncogene related to *SRC*, *FGR*, *YES*) is a member of the Src family kinases which are important in integrin-mediated cell adhesion and migration [31]. Activation of Fyn promotes the migration of squamous carcinoma cells [32]. *WASF3* (WAS protein family, member 3), also known as *WAVE3*, encodes a protein member of the WAVE family which mediates actin reorganization and cell movement. It has been reported that WAVE3, which is regulated downstream of PI3K, concentrates in the lamellipodia at the leading edge and mediates cell migration and lamellipodia formation [33].

The expression of *PVRL1* (1/1.97-fold) and *ACTG1* (1/1.05-fold) were shown to be suppressed by the applied dcEF. *PVRL1* (Poliovirus receptor-related 1), also called *nectin-1*, encodes a calcium-independent cell-cell adhesion protein. Nectin-1 plays a role in the organization of adherens junctions in epithelial and endothelial cells [34], [35]. *ACTG1* (Actin, gamma 1) encodes a cytoplasmic actin found in nonmuscle cells. Actins are well known to be involved in various types of cell motility, and maintenance of the cytoskeleton. The applied dcEF enhanced the expression of *ACVR1B*, *FYN*, and *WASF3*, which might in turn enhance the migration of CL1–5. It was suggested that EF-reduced adhesion (*PVRL1*↓) contributed to the enhanced motility. The suppression of *ATCG1* was minor compared to the change of the other genes. It has been shown that the migration rate of CL1–5 is enhanced when applying a dcEF to the cell culture region [8].

**Figure 5:**
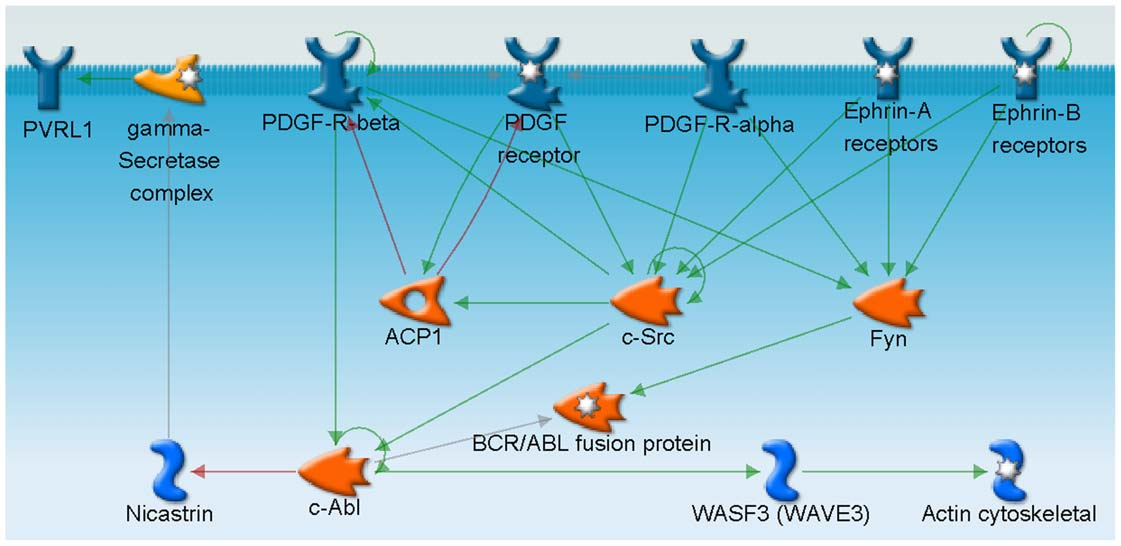
Protein-protein interaction between the products of the EF-regulated genes associated with adherens junction. The diagram showed that the EF up-regulated Fyn, ACP1, and c-Src might directly interact with two kinds of membrane receptors, PDGF receptors and ephrin receptors. Green arrow: positive effect; Red arrow: negative effect; gray arrow: unspecified effect.

We further investigated the relationship between the protein products of these six genes. The analytical tool MetaCore (GeneGo) was applied to build up the networks of the direct or indirect protein-protein interaction. The interactions and the subcellular localization of the proteins were shown in Figure 5. It was observed that Fyn, ACP1, and c-Src have direct interaction with two kinds of receptors, platelet-derived growth factor (PDGF) receptors and ephrin receptors [36]–[43]. *C-Src* was shown to be 1.3-fold up-regulated by dcEF in this study. PDGF receptors have been implicated to be activated by physical forces that alter the receptor conformation [44]. The networks suggested that PDGF receptors might be stimulated by the dcEF and then activated Fyn, c-Src and c-Abl, which influenced the activation of WAVE3 [45]–[47]. The activated WAVE3 subsequently regulated the reorganization of the actin cytoskeleton and thus influenced the cell morphology and motility [48]. The results also suggested that ephrin-A receptors and ephrin-B receptors might play roles in converting the electrical stimuli into biological signals. However, the gene expression change was not observed in PDGF receptors, ephrin receptors, and c-Abl in this study. The expression level and the role of these proteins in electrotaxis need to be investigated further.

Several EF-regulated genes were involved in the transcriptional regulation of telomerase RNA component gene *hTerc* (h_terc, BioCarta), which encodes the RNA component of human telomerase. The RNA component serves as a template for the telomere repeat [49]. Through the treatment of the EF, we observed that *NFYA* (Nuclear transcription factor Y, alpha) and *NFYC* (Nuclear transcription factor Y, gamma) were down-regulated with 1/1.16-fold and 1/1.27-fold, respectively. *NFYA* and *NFYC* encode two subunits of a trimeric complex NF-Y protein, which is an activator of the *hTerc* gene. The down-regulation of *NFYA* and *NFYC* suggested that the expression of *hTerc* may decrease due to the applied dcEF. Since there is no probe set for the *hTerc* gene in HG-U133 Plus 2.0 array, the expression of this gene was not shown in the microarray result.

The application of the dcEF to CL1–5 cells also altered the expression of important genes involved in the tight junction pathway (hsa04530, KEGG). Four correlated genes *F11R* (1.52-fold), *CTTN* (1.51-fold), *RAB13* (1.25-fold), and *EPB41* (1.23-fold) were shown to be up-regulated. *F11R* gene (F11 receptor), also called junction adhesion molecule-A (JAM-A), is an important regulator of tight junction assembly and cell migration [50]. *CTTN* gene (Cortactin) encodes a protein cortactin that regulates the interactions between components of adherent-type junctions. It organizes the cytoskeleton and cell adhesion structures of epithelia and carcinoma cells. Many studies have documented a role for cortactin in promoting cell motility and cancer invasion [51]. *RAB13* gene (member RAS oncogene family) encodes one of the small guanosine triphosphatases (GTPases) of the RAB subfamily, which is known to localize to the tight junction. This protein has been shown to play a critical role in regulating both the structure and function of tight junctions in polarized epithelial cells [52]. Rab13 protein is also shown to be activated during epithelial cell scattering-the process that includes the first steps of carcinoma invasion/metastasis [53]. The gene *EPB41* (Erythrocyte membrane protein band 4.1) encodes one of the 4.1 proteins called 4.1R. The 4.1 family proteins are components of the cortical cytoskeleton underlying the cell membrane. They contribute to the organization of cell polarity, adhesion and motility. They also affect transportation through the membrane and response to growth factors [54]. On the other hand, *PTEN* (1/1.19-fold) and *ACTG1* (1/1.05-fold) were shown to be down-regulated. *PTEN* is identified as a tumor suppressor and encodes a phosphatidylinositol-3,4,5-trisphosphate 3 (PtdIns(3,4,5)P3)- phosphatase. PTEN down-regulates intracellular levels of PtdIns(3,4,5)P3 and thus negatively regulates the PI3K/Akt pathway. Under the applied dcEF, PtdIns(3,4,5)P3 was reported to be polarized to the leading edge of differentiated HL60 cells migrating towards the cathode [16]. It is known that PTEN inhibits cell migration, spreading, and focal adhesions [55]. In brief, *F11R*, *CTTN*, *RAB13*, *EPB41*, *PTEN* and *ACTG1* were known to mediate cell motility and/or cytoskeleton organization, suggesting that they may play roles in the electrotactic response of CL1-5.

The confluent CL1-5 cell monolayers could be observed after the overnight incubation (∼20hrs) in the culture chamber of LEFC. It is reported that the development of high resistance electric seals takes 48-hour plating to reach the steady-state in tight junction formation [56]. Since the tight junction-associated genes were observed to be EF-regulated, we inferred that the dcEF may influence tight junction formation during the stimulation period. Thus, we further examined the gene expression of other major tight junction components, such as the transmembrane protein occludins and claudins, and the cytoplasmic ZO family. However, no significant difference was observed between the control group and the EF-treated group. The results suggested that dcEF might influence the tight junction assembly through regulating JAM-A instead of occludins, claudins, and ZO family in the transcription level.

To sum up, EF may influence the movement of CL1-5 by regulating the adherens junction and tight junction in transcription level. Many regulated genes in these two signaling pathways are known to correlate to cell migration. Except for *PTEN* and *ACTG1*, most of the genes have not been reported to be EF-correlated yet.

### Subcellular localization analysis

One known mechanism of electrotaxis is that membrane receptors redistribute under the applied dcEF and cause the asymmetric signaling of the cell [1]. It is also proposed that EFs may be transduced into mechanical signals by the mechanical torque exerting on the glycoproteins on the cell membrane [57]. Since the cell membrane proteins might be directly influenced by the dcEF, we would like to investigate their proportion in the products of the EF-regulated genes. About 18.2% of the significantly regulated genes (fold change >1.5 or<1/1.5) were observed to encode cell membrane protein. However, only 12% of randomly selected genes encoded cell membrane protein. The result indicated that a relatively high percentage of membrane proteins were significantly regulated by the dcEF in transcription level. The EF-affected membrane protein genes discovered in this work could be the candidates for the further glycomic study of electrotaxis.

### Biological function of EF-regulated genes

In addition to biological adhesion, we could see that the dcEF regulated the genes functioning in the biological processes such as biological regulation, cellular process, signaling, metabolic process, etc. (Figure 4). Several genes were known to participate in apoptosis, including the up-regulated genes *SRGN* and *TP53INP1* (Table 2), and the down-regulated genes *BIRC7*, *TRAF2*, *UNC5B*, and *GCLM* (Table 3). In addition, some other significantly regulated genes encoded the proteins correlated to G protein signaling, including the up-regulated genes *F2R* and *DOCK9*, and the down-regulated genes *GNA11*, *GPR156*, and *RAPGEF1*. It is known that G proteins are signal transducers that transmit chemical signals outside the cell, such as hormones, neurotransmitters, and chemokines, and cause changes inside the cell [58]. The result implied that G proteins may also play roles in transmitting mechanical signals in EF stimulation. Some other EF-regulated genes associated with G-protein signaling are observed in human dermal fibroblasts and adult epidermal keratinocytes [23], [24].

### Gene expression of reported electrotaxis-related genes/proteins

EGFRs have been reported to polarize in the cathode-facing side of lung cancer cell lines A549 and CL1-5 that migrated towards the cathode and the anode under the dcEF, respectively [7], [59]. It is also known that the EF-enhanced directional migration correlates well with the expression level of EGFR/ErbB1 in breast cancer cells. Transfection of MTLn3 cells and MDA-MB-435 cells with expression vectors for ErbB family members ErbB1, ErbB2 and ErbB3 also significantly enhance EF-induced migration [6]. In our microarray analysis, the expression change of *ErbB1* and *ErbB2* under the EF stimulation was not observed. The expression of *ErbB3* seemed to be down-regulated by the applied dcEF, since the signal strength of the control group (71.3 in average with all replicates >66.5) is larger than that of the EF-treated group (48.7 in average with all replicates <66.5).

Beta-4 integrin, encoded by *ITGB4*, is associated with alpha-6 integrin to be a receptor for the laminins. It has been reported that beta-4 integrin and EGF coordinately regulate the EF-mediated directional migration via Rac1 in human keratinocytes [15]. From the microarray analysis result, we did not see the regulation of *EGF*, *ITGB4*, and *Rac1* under the dcEF. However, *ITGB5* that encodes beta-5 integrin, another member of integrin family, showed significant up-regulation by the dcEF (1.55-fold, Table 2). Beta-5 integrin is associated with alpha-V integrin to be a receptor for the fibronectins. It has been reported that RNA interference knockdown of beta-5 integrin expression reduces cell migration *in vitro* and metastasis *in vivo* [60]. Our result suggested the positive correlation between the up-regulation of *ITGB5* and the enhanced migration of CL1-5 cells under the applied dcEF.

Rac and Cdc42 are GTPases that regulate lamellipodia and filopodia formation, respectively. They have been proposed to dominate the steering of growth cones cathodally in *Xenopus* embryonic spinal neurons. It has also been shown that RhoA, whose activation leads to growth cone collapse, elevates anodally in the EF-treated growth cones [17], [18]. Rho proteins regulate the dynamic assembly of cytoskeletal components for several physiological processes including cell motility. They are known to be involved in cell transformation and cancer metastasis. In our microarray analysis, we did not see the expression change of *Rac*, *Cdc42*, and *RhoA* under the dcEF. However, *DOCK9*, encoding a specific guanine-nucleotide exchange factor (GEF) that recognizes and activates Cdc42, was shown to be up-regulated by 1.94-fold (Table 2) [61]. Besides, *RhoJ*, which encodes another member of Rho family, seemed to be up-regulated by the EF treatment (accession # BC025770). For *RhoJ*, the signal strength of the EF-treated group (all replicates >66.5, 91.4 in average) is larger than that of the control group (all replicates <66.5, 32.4 in average). Recently, RhoJ is found to positively regulate endothelial motility and tubule formation [62].

EF-guided migration of rat hippocampal neurons is mediated by the activation of ROCK and PI3K. The inhibition of ROCK and PI3K decreased the directedness and the speed of the neuron migration [19]. In addition, electrical-signal-induced wound healing has been reported to be controlled by PI3Kγ and PTEN. Genetic disruption of PI3Kγ abrogates electrotactic migration of epithelial cells. On the other hand, deletion of the tumor suppressor PTEN enhances EF-directed keratinocyte migration [16]. In our study, *PI3K* and *ROCK* did not show obvious regulation by the applied dcEF. Nevertheless, *PTEN* was observed to be 1/1.19-fold down-regulated by the dcEF.

In summary, comparing the electrotaxis-related proteins reported in previous studies, some corresponding genes or their members in the families were observed to be regulated in the microarray analysis, but some were not. It is suggested that the polarization of proteins and the regulation of gene expression level both play roles in the mechanism of electrotaxis.

In addition, the calcium ions are reported to play important roles in the directed migration of *Dictyostelium* cells and the osteoblast-like cells [21], [22]. In this study, several EF-regulated genes were observed to be involved in the calcium signaling pathway (hsa04020, KEGG), including the up-regulated *F2R* (1.53-fold, Table 2) and *PLCB2* (signal intensities of the CR group <66.5), and the down-regulated *PHKB* (1/1.23-fold), *CALM1* (1/1.22-fold), *GNA11* (1/1.51-fold, Table 3), *ITPR2*, *GRIN2C*, and *ERBB3* (signal intensities of the EF group <66.5 for the last three genes). The result suggested that the calcium ions may also participate in the electrotaxis of CL1-5 cells.

### Microarray analysis in different EF-stimulated cells

Transcriptional response of human dermal fibroblasts (HDF-a) and human epidermal keratinocytes (HEKa) to the dcEF (100mV/mm, 1hour) has been studied through microarray analysis [23], [24]. Here we compare and discuss the EF-regulated gene expression changes in HDF-a cells, HEKa cells, and CL1-5 cells (Table 4). The significantly regulated genes were mostly different in these three types of cells, but some similarities and correlations were observed. For example, *PTEN* expression has been shown to be down-regulated in HDF-a cells and HEKa cells. Similar decrease of *PTEN* level was observed for CL1-5 cells. *MACF1* (microtubule-actin crosslinking factor 1) has shown down-regulation in HEKa cells. Similarly, decrease of *MACF1* was observed in CL1-5 cells. Nevertheless, some genes showed opposite responses to the EF stimulation. These differences were mainly observed between HEKa cells and CL1-5 cells. For example, *B4GALT6* (UDP-Gal:betaGlcNAc beta 1,4- galactosyltransferase, polypeptide 6) has shown EF-induced up-regulation in HEKa cells. In this study, *B4GALT6* was down-regulated in CL1-5 cells.

**Table 4:** Comparison of EF-induced gene expression changes in human dermal fibroblasts (HDF-a), human epidermal keratinocytes (HEKa), and human lung cancer cell line CL1-5.

Gene Name	Gene Symbol	Fold Change		
		HDF-a	HEKa	CL1-5
Phosphatase and tensin homolog (mutated in multiple advanced cancers 1)	*PTEN*	1.3	1.2	1.2
Ribosomal protein L10	*RPL10*	1.9	--	†
Microtubule-actin crosslinking factor 1	*MACF1*	--	1/1.5	1/1.3
UDP-Gal:betaGlcNAc beta 1,4- galactosyltransferase, polypeptide 6	*B4GALT6*	--	1.5	1/1.69
Alpha thalassemia/mental retardation syndrome X-linked (RAD54 homolog, S. cerevisiae)	*ATRX*	--	1/2	↑
WNK lysine deficient protein kinase 1	*WNK1*	--	1/1.8	↑
Directional migration		x [66]	cathode [67]	anode [8]

--: no reference.

† : the signal strength of the EF-treated group (two replicates >66.5) was larger than that of the control group (all replicates <66.5).

↑: the signal strength of the EF-treated group (all replicates >66.5) was larger than that of the control group (all replicates <66.5).

EF-stimulation condition: HDF-a and HEKa, 100mV/mm, 1hr, normal medium; CL1-5, 300mV/mm, 2hrs, serum-free medium.

Some differentially expressed genes in different cell types were shown to participate in the same signaling pathway. In HDF-a cells and CL1-5 cells, several EF-regulated genes were involved in TGF-β signaling pathway. In HDF-a cells, *THBS1* (thrombospondin 1), *BMP2* (bone morphogenetic protein 2), *DAF* (CD55 molecule, decay accelerating factor for complement (Cromer blood group)), *CD44* (CD44 molecule (Indian blood group)), etc. have been shown to be up-regulated [23]. In human lung cancer CL1-5 cells, *ACVR1B* (1.5-fold) and *ID1* (inhibitor of DNA binding 1, dominant negative helix-loop-helix protein, 1.3-fold) were observed to be up-regulated, while *LTBP3* (latent TGF-β binding protein 3, 1/1.6-fold) was down-regulated. It is reported that ID1 can be induced by BMPs in TGF-β signaling and in turn promotes cancer cell migration [63]. THBS1 and LTBPs affect the activation of TGF-βs, and THBS1 can be down-regulated by the inhibition of LTBP1 [64], [65]. BMPs and LTBPs are secreted proteins while THBS1 is an extracellular matrix protein. The correlation between HDF-a and CL1-5 in TGF-β signaling suggested that different types of cells may interact due to the EF stimulation.

Even under similar testing condition, HDF-a and HEKa show different transcriptional responses to the dcEF, suggesting that the dcEF has different influences on different cell types [23], [24]. Besides, it has been reported that dermal fibroblasts do not show significant migration under dcEF, epidermal keratinocytes migrate toward the cathode, and CL1-5 cells migrate toward the anode [8], [66], [67]. Thus, the differential transcriptional responses may correlate to the distinct electrotactic responses.

### Conclusion

A LEFC providing uniform dcEF was designed, fabricated, and used for sample collection in the electrotaxis study. The gene expression change of CL1-5 cells treated with the EF strength of 300 mV/mm for 2 hours was globally investigated by microarray analysis. Signaling pathway analysis of the EF-regulated genes showed that the dcEF may influence adherens junction, transcriptional regulation of telomerase RNA component gene *hTerc*, and tight junction of CL1-5 cells. Some up-regulated genes such as *ACVR1B*, *FYN* and *CTTN*, and some down-regulated genes such as *PTEN*, are known to be correlated with cell migration and cancer metastasis. The protein-protein interactions of adherens junction-associated EF-regulated genes suggested the participation of PDGF receptors and ephrin receptors in sensing electrical stimuli. Subcellular localization analysis of the significantly regulated genes showed that the applied dcEF affected the genes encoding cell membrane proteins in a relatively high ratio. In addition, the applied dcEF influenced many biological processes such as adhesion, signaling, and metabolic process. Some EF-regulated genes are known to be associated with cell apoptosis or G protein signaling. We further considered the gene expression of the electrotaxis-related proteins reported in previous studies. Some of the corresponding genes were observed to be regulated but some were not. It is suggested that the polarization of proteins and the regulation of gene expression both play roles in the mechanism of electrotaxis. Comparing the microarray analysis results in CL1-5 cells with that of the published studies in HDF-a cells and HEKa cells, we observed differential transcriptional responses between these three cell types. Some different EF-regulated genes in different cell types were shown to participate in the same signaling pathway, suggesting that different types of cells may interact under the EF stimulation.

## Materials and Methods

### Large electric-field chip (LEFC) fabrication and system setup

The LEFC chip consisted of several layers of poly-methylmethacrylate (PMMA) sheets, a layer of double-sided tape (8018, 3M), and a commercial 15cm cell culture dish (Corning) (Figure 1). The chambers, channels, and boundary of each part of the chip were designed by using AutoCAD (Autodesk). The patterns drawn on the software were transferred to a laser scriber (M-300, Universal Laser Systems) to cut pattern in each layer [68]. The patterned PMMA layers were then aligned and joined together to form a transparent PMMA chip by thermal bonding (120^o^C, 2hours). The PMMA chip, the patterned double-sided tape, and the 15cm cell culture dish were then assembled to form the LEFC with well-sealed channels and chambers.

The LEFC had connecting holes for the medium inlet/outlet and the agar salt bridges (Figure 1). Cells were cultured in the micro-chamber (the cell culture region). The width, length, and thickness of the micro-chamber were 24mm, 75mm, and 70 µm, respectively. For the electrotaxis experiment, a LEFC was integrated with a transparent ITO (Indium Tin Oxide) heater chip, two Ag/AgCl electrodes with phosphate-buffered saline (PBS) as electrolyte, two agar salt bridges (1.5% agar in PBS), a syringe pump, a DC power supply, an ampere-meter, and an inverted microscope (IX71, Olympus or TE2000-U, Nikon) (Figure 2). The distribution of dcEF in the micro-chamber was numerically simulated using a commercial software CFD-ACE+ (ESI Group) (Figure 3). The use of Ag/AgCl electrodes is essential because a simple design that uses platinum (Pt) wire as the electrodes introduces pH changes near the electrodes. More detail on the setup of the electrotaxis experiment can be found in the literature and our previous studies [8], [69], [70].

### Cell preparation and treatments

The lung cancer cell line CL1-5 acquired from Prof. Pan-Chyr Yang [25] was cultured in the complete medium consisting of Dulbecco's Modified Eagle's medium (DMEM, Gibco) and 10% fetal bovine serum (FBS, Invitrogen). CL1-5 cells were incubated in flasks (Corning) under 37°C and 5% CO_2_, and they were sub-cultured every 3–4 days. All experiments were performed with CL1-5 cells within 10 and 30 passages from the original source.

For the electrotaxis study, CL1-5 cells were cultured in the LEFC during the entire experiment. Before the experiment was started, CL1-5 cells were trypsinized from the flask and suspended in the culture medium with the density of 10^7^ cells per milliliter. Then the cells were injected into the micro-chamber manually and incubated for 4 hours for cell attachment. After that, the fresh culture medium was infused into the micro-chamber by a syringe pump at the rate of 200 µl/hr. The cells were incubated overnight under the culture medium flow. A transparent ITO heater chip below the LEFC (Figure 2) was controlled by a digital temperature controller (TTM-J4-R-AB, TOHO Electronics Inc.) to maintain the temperature (37+/−0.5^o^C) in the cell culture region. A temperature sensor (TPK-02A, TECPEL) was placed between the ITO chip and the LEFC for monitoring the temperature.

### Electric field stimulation

Before the dcEF was applied, the cell culture region was washed by serum-free medium (DMEM) with the flow rate of 200 µl/min for five minutes. Then the flow rate of serum-free medium was reduced to 200 µl/hr and the power supply was turned on. The galvanic current was conducted into the micro-chamber through the agar salt bridges, and it was monitored by an ampere-meter continuously. The agar salt bridges can separate the culture medium and the possible byproducts from the electrodes [1]. The strength of the dcEF was calculated based on Ohm's law [8]. The stimulation was lasted for two hours with the EF strength of 300mV/mm. For the control group, the experimental setup was the same except that the EF strength was 0mV/mm.

For gene expression analysis, the stimulated cells were collected from the LEFC immediately after the power supply was turned off. In brief, the serum-free medium in the LEFC was replaced by PBS with the flow rate of 200 µl/min. Then trypsin was injected into the micro-chamber and incubated for 1 minute in 37^o^C. Then the solution containing the cells was drawn out and mixed with the culture medium to stop the reaction of trypsin. The solution was centrifuged and the supernatant was removed. RNAlater (Ambion, Applied Biosystems) was then added to stabilize and protect RNA in the cells. The cells were stored at 4°C for later use.

### RNA isolation

Total RNA was isolated from the stored cells using RNaqueous kit (Cat# AM1912, Ambion, Applied Biosystems) according to the manufacturer's instruction. The quantity (µg/µl) and quality of total RNA was determined by Nanovue (GE Healthcare) and Agilent 2100 Bioanalyser (Agilent Technologies). For subsequent application, only samples with A260/A280 within 1.9∼2.2 were used.

### GeneChip hybridization

The microarray analysis was performed by using whole-genome Affymetrix GeneChip Human Genome U133 Plus2.0 Array, which represented 20722 genes from 54675 probe sets (based on the newest version (Release 31) of HG-U133 plus 2.0 annotation file). The GeneChips were processed at the Microarray Core of Institute of NTU Center for Genomic Medicine in National Taiwan University and the Affymetrix Gene Expression Service Lab of Institute of Plant and Microbial Biology in Academia Sinica. The procedure suggested by the manufacturer (GeneChip Expression Analysis Technical Manual rev5, Affymetrix) was followed. In brief, 10 µg total RNA was used for cDNA synthesis. Then, biotin-labeled cRNA was generated by *in vitro* transcription, using cDNA as templates. Finally, the biotin-labeled cRNA was fragmented. 10 µg labeled sample was hybridized to the GeneChips at 45°C for 16.5hours. The wash and staining were performed by Fluidic Station-450 and the GeneChips were scanned with Affymetrix GeneChip Scanner 7G.

### Microarray data analysis

Initial data analysis was performed by using the Affymetrix Microarray Suite v5.0 software, setting the scale of all probe sets to a constant value of 500 for each GeneChip. For further data treatment and analysis, the initial data were uploaded to the web server of composite regulatory signature database (CRSD) [71]. The initial data of the control group (3 biological replicates) and the EF-treated group (3 biological replicates) were batched and normalized with quantile normalization. To observe the different gene expression between the two groups, the analysis of variance (ANOVA) test was employed. After that, the signaling pathway analysis of the genes with statistical difference (p<0.05) was carried out by using CRSD, based on two databases Kyoto Encyclopedia of Genes and Genomes (KEGG, http://www.genome.jp/kegg/pathway.html) and BioCarta (http://www.biocarta.com/genes/index.asp). The degree of gene regulation is numerically expressed as the fold change, which is the quotient of the mean signal strength of the EF-treated group and that of the control group. Italic and standardized characters are used for representing the official symbol of genes and proteins, respectively.

### Real-time RT-PCR

The expression of the EF-regulated genes with higher fold changes was further validated by real-time RT-PCR. The primers were designed using the commercial software Primer Expression 3.0 (Applied Biosystems (ABI)), based on Affymetrix target sequences for the selected transcripts (Table S1). The specificity of the primer sequences was checked by NCBI BLAST. Total RNA (2 µg) was transcribed into cDNA with High Capacity cDNA Reverse Transcription Kit (Part# 4368814, Applied Biosystems). The reaction mixture consisted of Power SYBR Green PCR Master Mix (Applied Biosystems), 10ng cDNA, sterilized deionized water, and the forward and reverse primers (MDBio Inc.). The real-time PCR was performed using StepOne™ Real-Time PCR System (Applied Biosystems), following the protocol provided by ABI. StepOne Software v2.1 was used for the subsequent data analysis (Applied Biosystems). Relative quantification between the EF-treated group and the control group was carried out with the ΔΔCT method, using GAPDH as the endogenous house keeping control. All real-time RT-PCR expression values were determined from 2-3 independent biological experiments.

## Supporting Information

Movie S1 Response of CL1–5 to dcEF in serum-containing medium. The cells were treated with the EF strength of 200mV/mm for 4 hours in EFC. The serum-containing medium is the same as the culture medium.
(WMV)

Table S1 Forward and reverse primer sequences for real-time RT-PCR.
(DOC)
