# Supplementary material for: Gene Expression of Human Lung Cancer Cell Line CL1–5 in Response to a Direct Current Electric Field
Source: PLoS One. 2011 Oct 5;6(10):e25928. doi: 10.1371/journal.pone.0025928 (PMC3187831; doi:10.1371/journal.pone.0025928)
Supplement: Table S1 — Forward and reverse primer sequences for real-time RT-PCR. (DOC) [file pone.0025928.s002.doc]

Table S1 Forward and reverse primer sequences for real-time RT-PCR
